# Population Genetics, Evolutionary Genomics, and Genome-Wide Studies of Malaria: A View across the International Centers of Excellence for Malaria Research

**DOI:** 10.4269/ajtmh.15-0049

**Published:** 2015-09-02

**Authors:** Jane M. Carlton, Sarah K. Volkman, Swapna Uplekar, Daniel N. Hupalo, João Marcelo Pereira Alves, Liwang Cui, Martin Donnelly, David S. Roos, Omar S. Harb, Monica Acosta, Andrew Read, Paulo E. M. Ribolla, Om P. Singh, Neena Valecha, Samuel C. Wassmer, Marcelo Ferreira, Ananias A. Escalante

**Affiliations:** Center for Genomics and Systems Biology, New York University, New York, New York; Department of Immunology and Infectious Disease, Harvard School of Public Health, Boston, Massachusetts; Infectious Disease Program, The Broad Institute, Cambridge, Massachusetts; School of Nursing and Health Sciences, Simmons College, Boston, Massachusetts; Department of Parasitology, Institute of Biomedical Sciences, University of São Paulo, São Paulo, Brazil; Department of Entomology, The Pennsylvania State University, University Park, Pennsylvania; Liverpool School of Tropical Medicine, Liverpool, United Kingdom; Department of Biology, University of Pennsylvania, Philadelphia, Pennsylvania; Center for Infectious Disease Dynamics, The Pennsylvania State University, University Park, Pennsylvania; Department of Parasitology, Institute of Biosciences of Botucatu, State University of São Paulo, Botucatu, Brazil; National Institute of Malaria Research (Indian Council of Medical Research), Delhi, India; Department of Microbiology, Division of Parasitology, New York University School of Medicine, New York, New York; Institute for Genomics and Evolutionary Medicine, Temple University, Philadelphia, Pennsylvania

## Abstract

The study of the three protagonists in malaria—the *Plasmodium* parasite, the *Anopheles* mosquito, and the human host—is key to developing methods to control and eventually eliminate the disease. Genomic technologies, including the recent development of next-generation sequencing, enable interrogation of this triangle to an unprecedented level of scrutiny, and promise exciting progress toward real-time epidemiology studies and the study of evolutionary adaptation. We discuss the use of genomics by the International Centers of Excellence for Malaria Research, a network of field sites and laboratories in malaria-endemic countries that undertake cutting-edge research, training, and technology transfer in malarious countries of the world.

## Introduction

Whereas the first reference genome sequences of species of *Plasmodium*^1^–^3^ and *Anopheles*^4^ were generated by Sanger sequencing methods, advances in how genetic material is sequenced using so-called “deep sequencing” technology mean that sequence data can now be generated faster, more cheaply, and at much higher volume than before.^5^ The malaria community has embraced this revolution, and studies using deep sequencing methods have recently appeared that 1) probe the population genetics of *Plasmodium falciparum* on local^6^ and global^7^ scales, 2) enable comparative genomic studies between closely related species, for example, *Plasmodium vivax* and its sister taxon *Plasmodium cynomolgi* in the monkey malaria clade,^8^ and 3) generate additional reference genomes for 16 species of *Anopheles*.^9^ With the development of novel host deoxyribonucleic acid (DNA) depletion methods such as “hybrid selection,”^10^ we now stand poised to move genomics from the bench to the field.^10^

The rapid accumulation of new genome sequences of malaria parasites collected worldwide has also been accompanied by an increasing interest in understanding the patterns and mechanisms of genetic variation at the population level. “Population genomics” provides the tools to examine, for example, how new antigenic variants or drug-resistant strains emerge and spread in field parasite populations. A variety of DNA barcoding technologies are now available to sample subsets of genetic variants, such as single-nucleotide polymorphisms (SNPs), identified from whole genome sequencing (WGS). These genetic variants can collectively portray evolutionary changes in a population over time. In particular, changes in population structure can indicate changes in transmission, an important variable in malaria epidemiology. Tracking population structure signals to measure transmission as successful interventions deployed over time is a form of real-time epidemiology enabled by genomics technology.

Here we present some of the genomics, population genetics, and molecular evolution projects that are ongoing as part of several of the National Institutes of Health (NIH)-funded International Centers of Excellence for Malaria Research (ICEMR) initiatives. Our report is limited to projects involving genome-wide sampling and analyses, and encompasses genomic studies of parasite species *P. falciparum* and *P. vivax* as well as vector species of *Anopheles* (no human whole genome studies are currently underway at any of the centers).

## *Plasmodium* Population Genetics Using Genome-wide Markers

### Barcode/SNP genotyping.

Independent SNPs in a genome with a high minor allele frequency (MAF; defined as the frequency at which the least common allele occurs in a given population) can be used to assess the population structure of an organism, and this approach is being used by several ICEMR groups for assessing *Plasmodium* population structure. Population structure is inferred from patterns that indicate that the population is not undergoing random mating, such as the relative frequency of specific alleles in a population, linkage disequilibrium across otherwise physically unlinked loci, and the changes in those population parameters over time.^11^ Using high MAF SNPs from genome-wide sequencing, one can assess changes that a population may be undergoing due to external pressures, such as changes in transmission or prevalence of infection in the human population. These data can also be used to identify regions of the genome that are responding to, or associated with, phenotypic changes such as the acquisition of drug resistance. Finally, SNP genotyping data can be used to follow or track the effect of interventions on specific regions of the genome previously associated with, for example, drug resistance. Here we focus on the several ways that ICEMR groups are utilizing independent, high MAF SNPs to address changes in a parasite population over time, as a possible proxy for changing transmission dynamics. (SNP genotyping can be carried out using several different platforms that vary in the chemistries used, and each with its own advantages and disadvantages, but these are not discussed here.)

High MAF SNPs have been identified by screening available genome sequence data from multiple geographic settings to detect neutral, unlinked SNPs that are highly variable among the global parasite population. One set of these markers is the *P. falciparum* molecular barcode tool that surveys 24 unlinked SNPs (“24 SNP barcode”).^12^ This barcode has been used to demonstrate changes in parasite population structure coincident with a decline of malaria transmission in the west Africa ICEMR. The basic principle is that under high-transmission settings, there are a relatively large number of parasite genotypes that are being transmitted by mosquitoes to the human population, since cross-fertilization generates novel genotypes in the mosquito. As control interventions are deployed that successfully reduce transmission, the number of parasite genotypes in the population will be reduced, increasing the chance of self-fertilization. Eventually, as transmission decreases, the parasites within infected individuals will become genetically more similar. In the most extreme case, where only self-fertilization occurs, there will be evidence of clonal parasite genotypes being transmitted. Thus, determining the relatedness of parasites within a population can provide important data about malaria transmission, as well as enable evaluation of the success of interventions that reduce malaria transmission over time. In west Africa, the tool has been applied to show emergence of highly related parasites—including evidence of clonal parasite population structures—with increased deployment of malaria-reducing interventions.^13^

Several other ICEMR groups have also been deploying genotyping methods, including Malawi, southern Africa, southwest Pacific, and Latin America. For example, the southern Africa ICEMR (Zambia and Zimbabwe) has used the 24 SNP barcode to interrogate the impact of intervention strategies, detecting a decrease in parasite population signals corresponding with decreases in transmission (Sungano Mharakurwa and others, unpublished). In the Latin American ICEMR, this tool too has been used for outbreak investigation to show the clonal expansion of parasite populations in Panama.^14^

Several groups have been working on the development of a similar tool for *P. vivax*, distilling SNPs from *P. vivax* WGS data. One of these tools is based on markers that have a high MAF among a global parasite population and contains approximately 40 informative markers that can distinguish populations based on continental-level geography.^15^ A second approach has been to use SNP markers that separate parasites in close geographic proximity based on differences in allele frequencies (Alyssa Barry and others, southwest Pacific ICEMR, unpublished). Although these tools are still under development, they promise to provide useful genome-wide markers for asking important questions about *P. vivax* population structure.

### Microsatellite genotyping.

Despite the continuous development of novel molecular genotyping methods and high-throughput platforms, microsatellites (tandem repeats of motifs of two to six nucleotides) remain among the most popular and informative markers in population genetics.^16^ Several ICEMRs are using them in their studies, and although more details can be found in the accompanying Molecular Epidemiology supplement, we briefly review their use here as well.

Microsatellite abundance seems to correlate positively with genome adenine–thymine (AT) content, which is extremely high in *P. falciparum* (the average AT content reaches 95% in repetitive domains) but low in chromosome-internal regions of *P. vivax*. As a consequence, only ∼160 short repetitive sequences, many of them with features of classical microsatellites, have been characterized across the *P. vivax* genome.^1^ The extensive variation found in these genetic markers arises mainly from strand-slippage events during DNA replication. Observed microsatellite mutation rates (10^−3^ to 10^−4^ per locus per generation) result from the interplay between strand-slippage events and mismatch repair, which counteracts DNA slippage during replication.^17^ Thus, the high mutation rates of microsatellites allow their use whenever there is a need to understand population genetic patterns that emerged relatively recently and locally.^18^,^19^ On the other hand, SNPs and partial or whole genome sequence data are more appropriate for comparisons across the worldwide distribution of malaria parasites, since these patterns are the result of long-term processes (e.g., global patterns of population structure). The combined use of microsatellite markers with SNPs is a powerful population genomics approach that has been used in genome-wide association studies (GWAS), for example, to identify loci linked to artemisinin resistance, or multiple origins of drug-resistant haplotypes.^20^,^21^ Several ICEMRs are too using a combination of both markers for their projects, for example, the India ICEMR's planned *P. vivax* and *P. falciparum* GWAS studies use microsatellites to identify unrelated samples without extreme population structuring that are suitable for WGS.

## Sequencing Approaches to Examining Malaria Infections

Sanger sequencing (DNA sequencing based on incorporation of chain-terminating dideoxynucleotides during in vitro replication of a DNA molecule) has long been the workhorse of molecular biology efforts in malaria research. Although the first reference genome sequences of species of *Plasmodium*^1^–^3^ and *Anopheles*^4^ were generated by Sanger sequencing, deep sequencing is now almost exclusively used for whole genome studies, including probing the population genetics of *P. falciparum* on local^6^ and global^7^ scales, and comparative genomic studies between closely related species, for example, *P. vivax* and its sister taxon *P. cynomolgi* in the monkey malaria clade.^8^ Indeed, the use of deep sequencing is now encroaching on areas where Sanger sequencing used to rule, as exemplified below.

An example of deep sequencing methods providing extremely high resolution and sensitivity over Sanger sequencing is the use of the technology to examine within-host diversity of *Plasmodium* infections by amplicon sequencing.^22^ Briefly, this method uses targeted amplification of a polymorphic region in the *Plasmodium* genome followed by next-generation sequencing (NGS) so that hundreds or thousands of sequence reads are obtained that can be used to estimate the relative abundance of different clones in a single infection. Amplicon sequencing has been used to assess the genetic diversity in *P. falciparum* clinical samples, for example, using 454 pyrosequencing (454 Life Sciences, Branford, CT) to amplify the circumsporozoite (CS) gene locus, researchers could successfully identify 57 unique parasite haplotypes in 100 patient samples.^23^ Another study involving comparison of 454 amplicon sequencing and Sanger sequencing data for the CS gene revealed that the former could detect more variation than the latter and resolved the genetic diversity in complex infections much more sensitively.^24^

**Figure 1.**
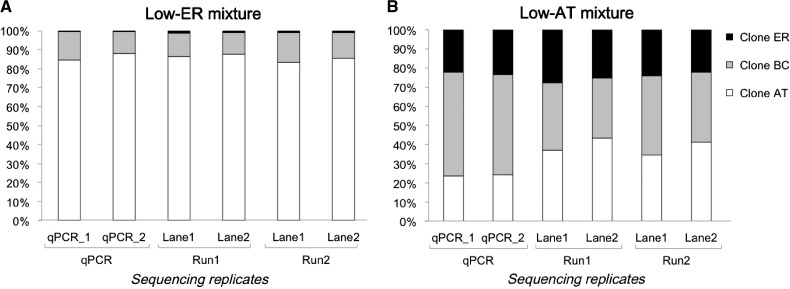
Quantification of multiclonal *Plasmodium chabaudi* mixtures using Ion Torrent amplicon sequencing and quantitative polymerase chain reaction (qPCR). Stacked plots representing proportion of *P. chabaudi* clones ER (black), BC (gray), and AT (white) obtained for two sets of parasite mixtures, shown in panels (**A**) and (**B**), containing low concentrations of the ER and AT clones, respectively. qPCR replicates are represented as qPCR_1 and qPCR_2, whereas sequencing replicates are grouped as Run1 and Run2 (biological replicates) and Lane1 and Lane2 (technical replicates).

Within the ICEMRs, the India group is using “selection differential” amplicon sequencing to identify the change in frequency of clones in *P. vivax* and *P. falciparum* samples from its three field sites in Chennai, Raurkela, and Nadiad. Preliminary experiments have used a *Plasmodium chabaudi* rodent malaria model, previously used to study aspects of mixed genotype infections, such as the dynamics of multiple infection,^25^ within-host competition in genetically diverse infections,^26^ and competitive release of drug resistance on drug treatment in mixed infections.^27^ *Plasmodium chabaudi* has several genetically distinct laboratory clones that can be distinguished based on sequence variation at the polymorphic antigen *Pcmsp1*.^28^ In a proof-of-principle experiment, artificial mixtures of DNA from several *P. chabaudi* clones grown in mice were generated, and the *Pcmsp1* locus amplified and sequenced to high coverage on an Ion Torrent sequencer (Life Technologies Corporation, Carlsbad, CA). Sequencing reads could be aligned uniquely to the *Pcmsp1* sequence of each laboratory clone, and were used to quantify the relative frequency of each clone in the artificial mixtures. Technical and biological sequencing replicates were found to be highly reproducible, and independent estimates of the relative abundance of different clones obtained using quantitative polymerase chain reaction (qPCR) confirmed the accuracy of the method (Figure 1

).

The use of amplicon sequencing is well suited to interrogate field samples, as it requires much less DNA than WGS, and can be obtained from filter paper blood spots. However, the use of NGS is not without challenges. For example, as sequence reads are prone to error, it can be difficult to accurately quantify the genetic diversity in a heterogeneous sample. A few computational methods are available for performing global haplotype reconstruction by clustering deep sequencing reads based on sequence variation in the presence or absence of a reference genome (see References 29, 30). The India ICEMR is also developing statistical methods to provide estimates of the power of the approach in detection of low-frequency variants and estimation of population structure, while considering the various sources of errors.^22^

## WGS Projects

**Figure 2.**
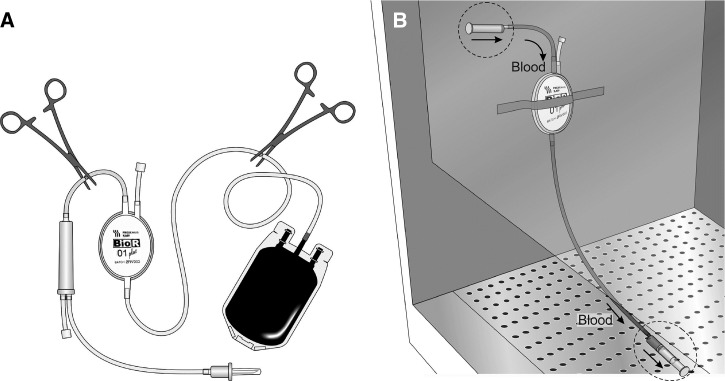
A simple and efficient filtering procedure to remove leukocytes from small volumes of *Plasmodium*-infected venous blood. The left panel (**A**) shows how to prepare commercially available leukocyte depletion filters (Fresenius Kabi BioR 01 Plus) for removing leukocytes. Note that the tubing must be cut with a scissor as indicated, to remove the storage bag and the adaptor from the filtering device. The right panel (**B**) shows how the adapted filters are used, in a laminar flow safety hood. A 10-mL syringe is used to apply acid citrate dextrose (ACD) treated blood samples, whereas a second 10-mL syringe is adapted to the end of the tubing to recover the filtered (leukocyte-depleted) material. After the filtering procedure, the leukocyte depletion filter is washed extensively with RPMI 1640 medium to recover red blood cells that have been retained in the tubing.

Human, *Anopheles*, and *Plasmodium* genome-scale studies can present many challenges, from the retrieval of sufficient high-quality biological material, to storage and manipulation of large sequence data files, and data analysis and visualization. In particular, low parasitemia and human DNA “contamination” in clinical samples can complicate WGS of *Plasmodium* field isolates. Most current protocols for *Plasmodium* clinical sample processing include CF11 column filtration to remove human leukocytes (the source of contaminating host DNA), or for *P. vivax*, 48 hour schizont maturation ex vivo to increase parasite DNA.^31^ The Amazonia ICEMR has found a simple and efficient alternative for removing contaminating host DNA from relatively small volumes (10–50 mL) of venous blood, by adapting commercially available leukocyte depletion filters (Fresenius Kabi BioR 01 Plus or Max) that are commonly used in clinical hemotherapy. The simple filtering procedure described in Figure 2

produces 88% red blood cell recovery and ∼94% of the sequence reads mapping to the *P. vivax* Salvador I reference genome. In contrast, the India ICEMR is using “hybrid selection” to enrich parasite material from *P. falciparum*^10^ and *P. vivax*^32^ samples used for whole genome studies.

WGS of *Plasmodium* genomes is complicated by extreme AT-rich regions, particularly in the case of *P. falciparum* (80.6% AT), although the subtelomeric ends of *P. vivax* chromosomes are also highly AT biased. This results in low sequencing coverage of such regions, making genome assembly and variant calling challenging, although in *P. vivax* both of these processes are somewhat easier.^33^ Another difficulty in sequencing *Plasmodium* clinical samples is posed by polyclonality, that is, mixed genotype infections. The presence of multiple genotypically distinct parasites in a single patient sample can result in an overestimation of the level of genetic diversity in a parasite genome, and potentially confound subsequent population genetic analysis.^7^ It is important to understand and consider these issues when analyzing WGS of *Plasmodium* field isolates. A summary of the WGS projects being undertaken as part of the ICEMR network is shown in Table 1 and described below.

### Mosquito WGS.

#### Anopheles gambiae 1000 Genomes Project.

The east Africa ICEMR has contributed specimens to the *An. gambiae* 1000 Genomes Project, an international consortium of northern and southern partners that uses Illumina-based WGS to develop a detailed understanding of genetic variation in wild-caught populations of the major malaria vectors within the *An. gambiae* complex. The initiative is part of the MalariaGen Network (http://www.malariagen.net/projects/vector/ag1000g) and sequencing is performed at the Wellcome Trust Sanger Institute. Samples have been obtained from across the species range of *An. gambiae* and *Anopheles coluzzii*, with some collections also including *Anopheles arabiensis.* The east Africa ICEMR has contributed specimens from Tororo, eastern Uganda, a region with a high entomological inoculation rate^34^ and extensive insecticide resistance.^35^,^36^ Data from 103 female *An. gambiae* mosquitoes are currently available (http://www.malariagen.net/data/ag1000g-phase1-preview), and the data may be explored using a purpose-built, highly interactive web browser (http://www.malariagen.net/apps/ag1000g/phase1-preview/static/main.html). In later phases of the project, WGS data from sympatric *An. arabiensis* will be included to investigate the phenomenon of interspecific introgression. Data from a 1,536 SNP Goldengate microarray have demonstrated that there is extensive contemporary gene flow between the two species in this region,^37^ and it will be determined if there is any evidence for the transfer of adaptively advantageous alleles as has been observed between *An. gambiae* and *An. coluzzii*.^38^ In due course, it is likely that other ICEMR partners will join the consortium as the geographic and taxonomic range of the sequencing effort is extended.

#### Population genomics of Anopheles darlingi in Brazil.

Other groups have used partial genome sequencing to sample the diversity of *Anopheles*. For example, to characterize the genetic diversity of *An. darlingi* populations collected in different rural settlements in Acre State in the Amazon Forest in Brazil, researchers in the Amazonian ICEMR have used ddRAD-Seq to generate a catalog of ∼150,000 genome-wide 100-bp tag sequences from 90 individual mosquitoes. ddRAD-Seq (restriction-site associated DNA sequencing) uses a pair of restriction enzymes to generate fragments of a genome that are then sequenced to produce genetic markers for population genomic surveys, and is a “reduced representation” method^39^ that enables sampling of large, complex genomes such as *Anopheles* without sequencing the whole genome.^40^,^41^ Population genetic analysis of different populations with this catalog of sequences is beginning to reveal that 1) genetic diversity of *An. darling* populations appears to be inversely proportional to deforestation; and 2) populations separated by 100 km, indistinguishable in terms of microsatellite diversity, can be differentiated by the SNP polymorphisms identified in the ddRAD-Seq tags.

### *P. falciparum* WGS.

#### WGS of drug-resistant P. falciparum from patients at the China–Myanmar border.

Genome-based approaches to determine the molecular bases of antimalarial drug resistance are a powerful tool. With the recent detection of artemisinin-resistant parasites in multiple countries of the Greater Mekong subregion of southeast Asia,^42^,^43^ the southeast Asia ICEMR is focusing its drug resistance monitoring and research efforts on parasite populations along the China-Myanmar and Thailand-Myanmar borders, which have unique antimalarial drug use histories. Using parasites procured from clinical cases of malaria from these regions, they are using GWAS to identify genes or genomic regions associated with altered sensitivities to artemisinin and artemisinin combination therapy (ACT) partner drugs. With an aim of obtaining complete genome sequences from ∼150 single parasite isolates, this study will provide an unprecedented opportunity to perform population genomic studies and GWAS in the deciphering of artemisinin and ACT drug resistance mechanisms.

#### WGS of P. falciparum from cerebral malaria patients in India.

The identification of parasite genetic determinants of cerebral malaria (CM), the most severe complication of *P. falciparum* infection, has been unsuccessful so far. This has been mainly attributed to the highly diverse parasite population in hyper-endemic areas where the analyses were carried out, and to the variation in host susceptibility among patients, which is likely to play an important role in the progression of the disease. In addition, the clinical diagnosis of CM is not straightforward, exemplified by a study conducted in Malawi that revealed ∼25% of pediatric patients with World Health Organization-defined CM had a non-malaria cause of death.^44^ This misdiagnosis may have represented an important bias in the selection of “false” CM isolates of *P. falciparum* in previous genetic studies. The India ICEMR is evaluating the genetic basis of malaria disease phenotype using WGS of clinical isolates collected from adult patients as part of an ongoing study of CM pathology by magnetic resonance imaging (MRI; see accompanying Pathogenicity article). Since falciparum malaria is complex and results in a broad spectrum of disease in adults, the project focuses on comparing the genomes of *P. falciparum* isolates from CM and uncomplicated malaria (UM) patients, at the opposite ends of the disease severity spectrum. These patient categories are carefully defined as part of the MRI study, and the occurrence of CM is assessed by combining the World Health Organization criteria with a systematic ophthalmic examination for the presence of malaria-associated retinopathies, a clinical feature associated with the severity of malaria, mortality, and duration of coma.^45^,^46^ Collection of isolates during the same malaria season in a mesoendemic transmission area in combination with population genetics will reduce confounding factors of population structure and reduce the sample selection biases to a minimum. *Plasmodium falciparum* DNA is being extracted directly from venous blood samples and hybrid selection used for parasite DNA enrichment, as described above.^10^ WGS on the Illumina HiSeq2500 platform (Illumina Inc., San Diego, CA) will yield up to 130× coverage of the *P. falciparum* genome, and data processing will include quality control, sequence alignment, and variant discovery based on the *P. falciparum* 3D7 reference sequence.

### *P. vivax* WGS.

Although *P. falciparum* can be cultured in vitro, so that laboratory-adapted clones can be generated from patient infections and phenotyped for important traits such as drug resistance, no such luxury is available to the *P. vivax* researcher. Thus, genomics is a key methodology that can provide an enormous amount of information about *P. vivax* for relatively little input. Below we describe several of the ICEMR efforts in this area.

#### Cross-ICEMR global genetic diversity map.

One of the largest cross-ICEMR efforts is being undertaken by the India ICEMR as part of a collaboration with the Broad Institute sequencing center. Approximately 180 clinical *P. vivax* isolates collected from vivax-endemic regions under ICEMR purview have been sequenced at New York University (NYU) and the Broad (Table 2 ) as part of a project to develop a global genetic diversity map of the species (see https://olive.broadinstitute.org/projects/plasmodium_vivax_hybrid_selection). Each isolate has been verified by species-specific PCR, as well as genotyped at one or several loci to determine if infections were multiclonal, and many isolates are also linked with metadata. As this project progresses, analysis will turn to bioinformatic methods to assess mixed genotype infections that will overlap with the molecular data to establish a firm understanding of each isolate's history of infection. Additional goals are to use the variant calls and sequence data to generate phylogenetic histories and assess the impact of natural selection on this global population of *P. vivax.*

#### Whole genome studies of local populations.

Although the study described above will provide a global overview of diversity and population genomics, several ICEMRs are undertaking more localized whole genome analyses. For example, researchers in the India ICEMR are planning WGS of tens of *P. vivax* isolates in their three field sites for a genetic diversity map of the species in India, as well as identifying phenotypes of interest such as severe vivax infections that could be interrogated in a GWAS study. In the Amazonia ICEMR, researchers are analyzing sympatric *P. vivax* from the ICEMR field site of Remansinho (Acre, Brazil) where the species is responsible for about 85% of malaria cases. In Remansinho area, *P. vivax* transmission has declined substantially over the last 3 years and *P. falciparum* has not been found since March 2011.^47^ The genomic study of sympatric isolates from an area of low malaria endemicity is particularly interesting in this case due to three factors. First, since these isolates are on the whole more closely related, they are suitable for revealing recent selective pressure events. Second, sympatric isolates are less likely to present population substructure, whose genetic effects (e.g., linkage disequilibrium or particular allele frequency patterns) could be confused with the signature of natural selection. Third, as shown for *P. falciparum* isolates from Amazonian Peru,^48^ sympatric parasite samples are likely to comprise one or both parental genotypes and the recombinant progeny of natural genetic crosses, allowing for a preliminary evaluation of the relative role of mutations and recombination to generate genome-wide diversity in local parasites. With that in mind, the team has recently sequenced the complete nuclear genomes of nine *P. vivax* genomes collected in a well-defined area (radius, 100 km) around Remansinho, in the western Amazon Basin of Brazil, close to the border with Bolivia (Table 1).

#### Comparative genomics of P. vivax.

The origin of *P. vivax* as a species comprises a series of complex evolutionary events.^49^–^51^ The lineage leading to *P. vivax* is part of a diverse monophyletic group of nonhuman primate malaria parasites that radiated in southeast Asia,^51^ and among them, the macaque parasite *P. cynomolgi* seems to be the closest known species to *P. vivax* in that clade. This species is being used in the ICEMRs as part of comparative genomic studies to better understand the polymorphism in *P. vivax*.^8^ The emerging patterns indicate that there is extraordinary polymorphism in *P. vivax* multigene families that emerged after the *P. cynomolgi*–*P. vivax* split.^52^ These studies are allowing us to carefully explore fissures in *Plasmodium* genomes such as the discovery that the *msp-3* gene family is not homologous between *P. vivax* (and related species) and *P. falciparum*,^52^ and to describes how natural selection acts on the *P. vivax* genome.^53^ The adaptive value of such patterns is a matter that will be explored in the context of *P. vivax* population genomics investigations, and these studies are further stressing that discoveries in *P. falciparum* do not immediately translate into *P. vivax*.

## Implementing Genotyping and Genomics Technology in a Field Setting

Three general challenges must be met to successfully deploy and use genotyping or genomics technology in field-based settings. First, the purchase of durable and practical equipment is key, and this decision is based on a number of factors including cost, equipment versatility (e.g., ability to perform different types of assay), and flexibility of the technology for other purposes (e.g., the Luminex platform can perform immunology-based assays). Once the major use has been ascertained, other considerations including the cost per assay, throughput capabilities, and the sensitivity of the assay in terms of limits of detection, must be considered. Second, there is the issue of purchasing reagents and sustainability of the technology in-country. Reagent procurement can be facilitated in one of two ways: either with collaborators assisting the purchasing, or with the endemic country scientist implementing their own procurement system. These requirements often come with an additional cost burden since shipping such reagents directly can be extremely expensive; there may be a cold chain requirement, and access to reagents may be sporadic. Machine maintenance is another issue, since repairs and preventative maintenance may also be limited. One model that has been successful for several ICEMRs is the utilization of collaborations for procurement and shipping of reagents and supplies and even for swapping out machines for repair. Leveraging these types of relationships also usually has additional cost benefits for purchase of reagents. The third challenge is training in the transfer of the technology, study design, data analysis, and data interpretation, and here the ICEMRs have been highly productive because of their “network” of interactions. For example, workshops have been held in concert with the annual ICEMR meeting (e.g., the Workshop on Population Genetics in Lima, Perú held in August 2014, and the Workshop Using Protein Array Data in Guilin, China held in August 2013), or for those in close proximity there have been joint ICEMR workshops (e.g., the Zambia January 2013 genotyping workshop held jointly between the southern and west African ICEMRs). In addition, the eukaryotic pathogen database EuPathDB has focused on including ICEMR members in its workshops held close to ICEMR sites. These include workshops in Bogota, Colombia (July 2013) and Singapore (February 2014). EuPathDB provides an online compendium of its workshops material (e.g., see here http://workshop.eupathdb.org/singapore/2014/ for the 2014 Singapore workshop material). Thus, the ICEMR network has promoted training opportunities that move malaria-endemic country scientists toward independence and sustainability of these skills, so that they can better address malaria control and elimination issues.

### Developing small-scale field-based genotyping capability.

Several ICEMRs have invested in training in generation and analysis of genotyping data—including SNPs, copy number variants, and microsatellites—used to understand changes in population structure of both parasite and mosquito. Many genotyping technologies use PCR-based methods to amplify a region surrounding the variant locus, and differ by the marker type and the technology platform used to assay for it. Examples of technologies that are being used by ICEMR sites in the field include: 1) PCR amplification followed by sequencing or restriction endonuclease digestion,^54^–^56^ 2) TaqMan genotyping,^12^,^57^–^59^ 3) high resolution melting,^60^,^61^ and (3) ligase detection reaction-fluorescent microsphere assays (e.g., the Luminex platform^62^). Although the chemistry underpinning these technologies varies, and consequently each has its own advantages and disadvantages, basically all of these and other technologies may be used to identify genetic variants.

### Developing next-generation sequencing expertise in malaria-endemic countries.

Next generation sequencing has revolutionized the field of biomedical research over the past decade, and while the leading sequencing platforms were initially tailored toward large-scale applications, recent technical improvements have led to the development of modestly priced benchtop instruments with fast turnover rates aimed at small-scale laboratories. Three of the most widely used benchtop instruments are the Roche 454 GS Junior (454 Life Sciences, Branford, CT), Illumina MiSeq (Illumina Inc., San Diego, CA), and the Ion Torrent Personal Genome Machine (PGM) (Life Technologies Corporation, Carlsbad, CA). A performance comparison of these sequencing platforms based on technical specifications, data quality, and throughput, as well as a review of their potential applications, setup, and running costs has been described in detail elsewhere.^63^,^64^ Although there are advantages and disadvantages associated with each platform, depending on the type of applications being considered, they are closely matched in terms of utility and ease of workflow.^65^ Therefore, the choice of platform often depends on other factors, such as existing infrastructure, available finances, and personal experience of users.

#### A view from India.

The India ICEMR chose to purchase the Ion Torrent PGM platform (with the fastest throughput, shortest run time, and least expense) for amplicon sequencing and *P. vivax* whole genome re-sequencing of Indian samples in Delhi, with a sister machine at NYU. Thus protocols can be tested at NYU and directly transferred to the machine in Delhi. The instrument comes with the Torrent Suite software and browser, a web-based interface for planning, monitoring, viewing, and processing results from a sequencing run, including sequence alignment, coverage analysis, and variant detection, which can be used to create a custom analysis workflow. The ease of use and minimum information technology expertise required to operate the data analysis tools on the PGM machine make it an attractive option for endemic country scientists without advanced training in bioinformatics, and without access to a high-performance compute cluster. Currently, the PGM in Delhi has been run successfully several times using clinical samples, and will be running continuously in 2015.

#### A view from Brazil.

High-throughput sequencing facilities are becoming more common in Brazil, and becoming more efficient as they accrue experience. Nevertheless, due to local bureaucratic, tariff/tax, and market issues, genome sequencing can be significantly more expensive than in other countries, and weeks or even months can pass before reagents are available. For that reason, many researchers use the services of foreign companies or facilities for high-throughput sequencing—which solves some of the problems, but still suffers from bureaucracy and is therefore impractical for larger studies. Challenges related to the analysis of high-throughput data are also present in Brazil, for example, incipient computational infrastructure for bioinformatics at universities, and a relative scarcity of students, technicians, and researchers possessing the required quantitative and computational skills to deal with large-scale biological data.

Nonetheless, members of the Amazonia ICEMR have used the Ion Torrent PGM and Ion Proton platforms in Brazil and analyzed the resulting sequence at the National Laboratory for Scientific Computing in Petrópolis, Rio de Janeiro. Reads of ∼200 bases in length, and sequencing depths of 50- to 80-fold coverage are being achieved, and SNP analysis is being performed using GATK,^66^ and assembly using Newbler (Roche 454), for comparison with the *P. vivax* Salvador I reference genome and with other less complete *P. vivax* genomes. Future analysis of the nine Brazilian sympatric genomes will include investigations of genome structure and evolution, recombination, population structure, selective pressure, and phylogenetics.

## Genome Databases

The eukaryotic pathogen database EuPathDB (http://eupathdb.org) is an NIH-funded bioinformatic resource center.^67^ The goal of this free and widely available online resource is to integrate a variety of large-scale datasets from eukaryotic pathogens and their hosts, and facilitate data interrogation in an easily accessible system. Data types already available in these include genome sequence and annotation, proteomic data (including quantitative), transcriptomic data (RNAseq, microarray), epigenomic data (ChIP-Chip and ChIP-seq), population data based on high-throughput sequencing, and phylogenetic data. The ability to effectively integrate population data necessitates the collection of useful metadata from the data provider that is interoperable between different providers, allowing questions to be asked not only about a specific dataset but also across datasets from diverse groups or projects. EuPathDB has invested a significant effort in ensuring data integration by mapping metadata to already established ontologies such as the Ontology of Biomedical Investigation (see accompanying manuscript describing data management of ICEMR projects).

**Figure 3.**
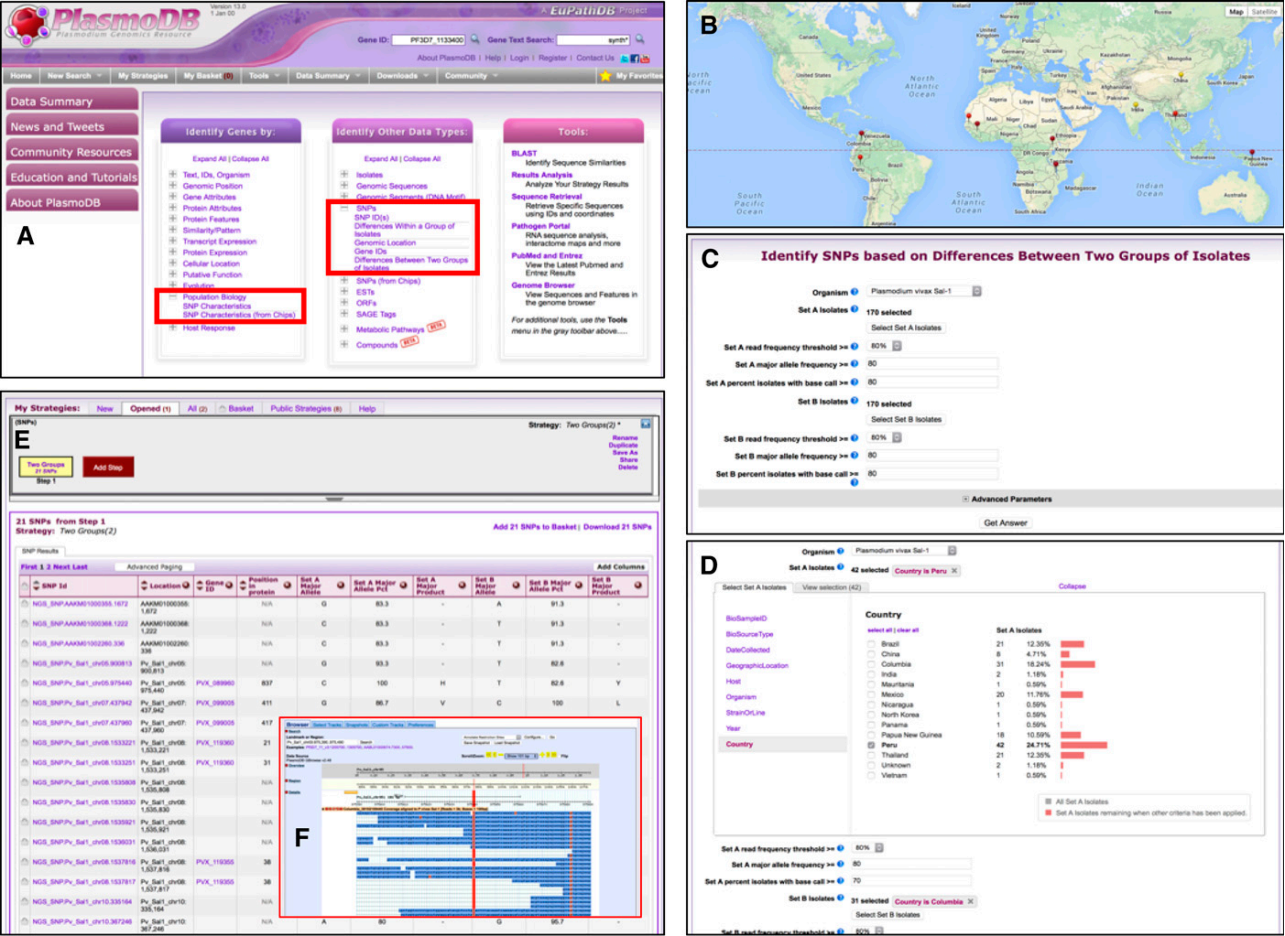
Screen shots from PlasmoDB illustrating methods to access single-nucleotide polymorphism (SNP) data from International Centers of Excellence for Malaria Research (ICEMR) projects. (**A**) PlasmoDB home page showing where SNP data can be accessed (red rectangles). (**B**) Map of the geographic distribution of sequenced *Plasmodium* isolates from ICEMR locations. (**C**) Sequenced isolates can be compared in PlasmoDB using the search “Identify SNPs based on differences between groups of isolates.” (**D**) Metadata characteristics such as geographical location can be leveraged to identify SNPs that differentiate isolates from Peru and Colombia. (**E**) Results are returned in a table containing the location of the SNP and various SNP statistics. (**F**) The sequence alignment around any SNP can be visualized in the PlasmoDB genome browser.

**Figure 4.**
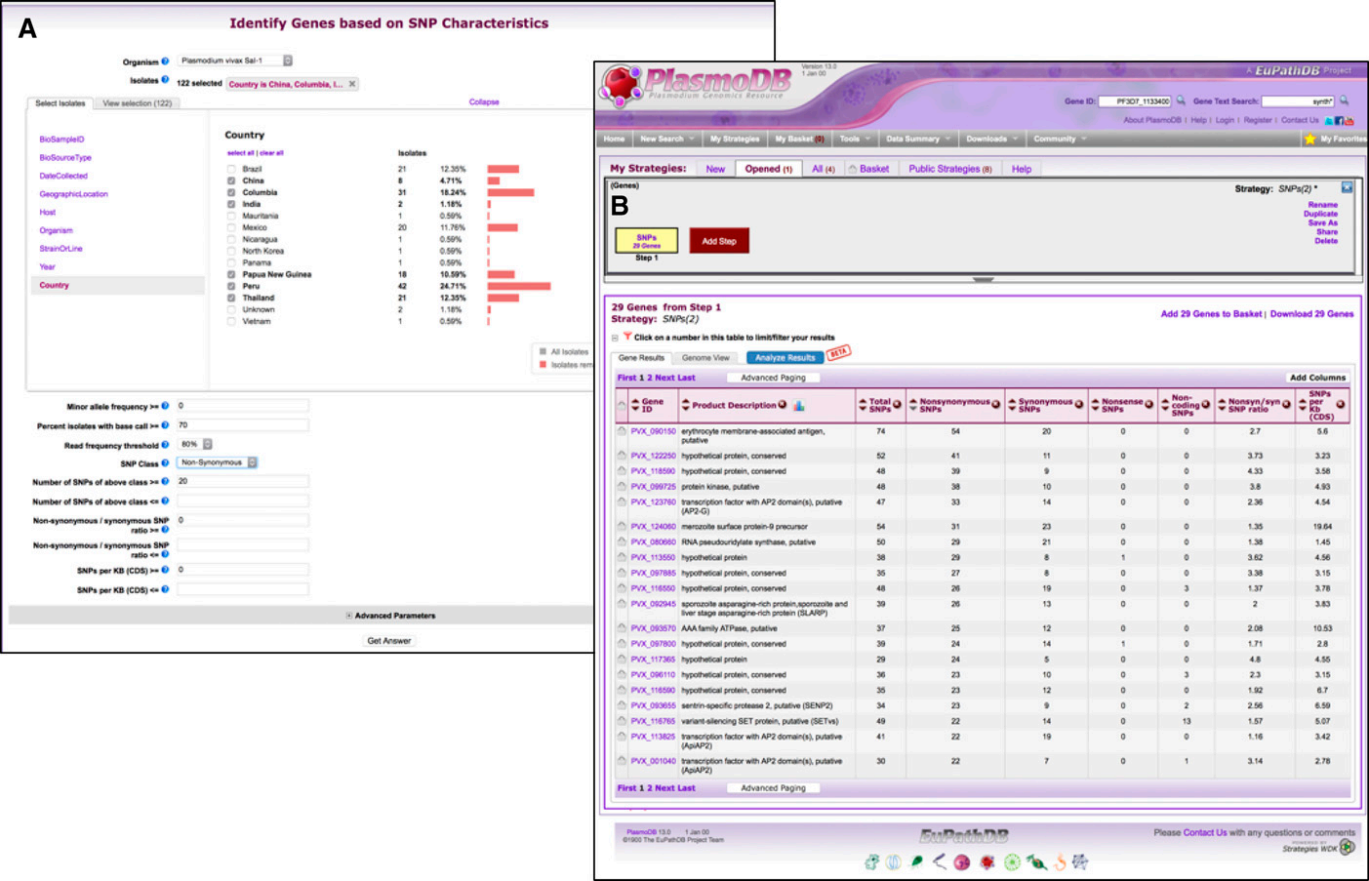
Screen shots from PlasmoDB depicting a search for genes containing at least 20 non-synonymous SNPs based on data from ICEMR isolates. (**A**) The search for genes based on SNP characteristics allows filtering of isolates based on metadata, such as geographic location, and defining the type of SNPs of interest. (**B**) Results are returned in a table that shows which genes contain the SNPs of interest.

PlasmoDB (http://plasmodb.org) and HostDB (http://hostdb.org) are components of EuPathDB that focus on integrating data from *Plasmodium* species and their host organisms. Importantly, the ability of these databases to accommodate high-throughput sequencing data from field studies along with their associated metadata allows sophisticated filtering and searching tools, making both databases suited for cross-ICEMR data interrogation. High-throughput sequencing data from ICEMR projects has been integrated into PlasmoDB and can be searched using gene-specific searches that identify genes with user-defined SNPs, or searches that identify SNPs regardless of their presence in genes (Figure 3

). Genes may also be searched based on SNP characteristics identified from ICEMR sequencing data (Figure 4

).

## Figures and Tables

**Table 1:** List of *Anopheles* and *Plasmodium* species being sequenced as part of the ICEMR initiative, and the main research questions being asked

Species	ICEMR	Country	Sequencing approach	Number of genomes	Main research questions
Vector					
*Anopheles darlingi*	Amazonia	Brazil	ddRAD	NK	Population genetics with regard to deforestation
*Anopheles gambiae*	East Africa	Uganda	WGS	115	Population genetics
*Anopheles arabiensis*	East Africa	Uganda	WGS	82	Interspecies introgression
Parasite					
*Plasmodium falciparum*	Southeast Asia	China-Myanmar	WGS	> 150	Drug resistance
*P. falciparum*	India	India	WGS	30	Genetic determinants of cerebral malaria
*Plasmodium vivax*	Latin America, southeast Asia, Amazonia, southwest Pacific, India	Brazil, Colombia, Papua New Guinea, Peru, India, China, Mexico, Thailand	WGS	∼180	Global genetic diversity map
*P. vivax*	India	India	WGS	> 30	Genetic diversity map, GWAS of traits
*P. vivax*	Amazonia	Brazil	WGS	9	Sympatric population
*P. vivax*	Amazonia	Peru	WGS	NK	Methods for enriching parasite-derived DNA in field samples

ddRAD = double-digest restriction associated DNA; DNA = deoxyribonucleic acid; GWAS = genome-wide association studies; ICEMR = International Centers of Excellence for Malaria Research; NK = not known; WGS = whole genome sequencing.

**Table 2:** Geographical location and number of *Plasmodium vivax* isolates from ICEMR sites sequenced for generation of a global genetic diversity map

Country	ICEMR	No. of isolates	Comments
Brazil	Amazonia	20	Sequenced at Broad
China	Southeast Asia	8	Sequenced at Broad
Colombia	Latin America	31	Sequenced at Broad
India	India	9	Sequenced at NYU
Mexico	NA	20	Sequenced at Broad
Peru	Amazonia	47	Sequenced at Broad
Papua New Guinea	Pacific	23	Sequenced at Broad
Thailand	NA	20	Sequenced at Broad
Nicaragua, Panama, Thailand, Vietnam	NA	4	Monkey-adapted strains from MR4
Total: 11 countries	5 ICEMRs	182 isolates	–

Broad = Broad Institute of Harvard and MIT (Massachusetts Institute of Technology); MR4 = Malaria Research and Reference Reagent Resource; NA = not applicable; NYU = New York University.
